# Theory Concerning the Cause of Diabetes

**Published:** 1849-01

**Authors:** 


*Theory concerning the Cause of Diabetes.*—At a meeting of the Academy of Medicine, M. Mialhe read a paper on Diabetes. The author assigns as a cause of saccharine urine, and the disorders consequent upon its secretion, a want of sufficient alkalinity in the fluids of the body. To him the cause of diabetes is not a peculiar agent which gives diabetic patients the faculty of transforming certain alimentary substances into sugar, which latter entering the torrentof the circulation is eliminated by the urine; but he maintains, first, that the transformation of amyloid substances into sugar is not peculiar to diabetic patients; that it is not an accidental phenomenon, but that it is, on the contrary, a necessary part of the digestion and assimilation of food. Secondly, that this transformation is brought about by the agency of a special ferment, which the author has discovered in the salivary glands of all animals, and which exercises a specific action on fæculent substances similar to the action of diastose (the active principle of malt) on starch, whence he calls this ferment *animal diastose.* Thirdly, amyloid substances must, in all animals, without exception, be converted into sugar under the influence of that animal diastose, in order to become fit for absorption and assimilation. But, says the author, what becomes of this sugar? It must participate in nutrition, and in order to do this it must suffer decomposition in the circulating fluids, for in the normal state it cannot be detected in any of the secretions. When it passes unaltered through the kidneys, it may be inferred that some powerful cause has prevented its decomposition, and thereby rendered it unfit for assimilation. This is, then, an abnormal and pathological occurrence which may be regarded as the consequence of the perturbation of another order of chemical phenomena; and this perturbation consists in a want of alkalinity in the fluids of the animal economy. Here the author, grounding himself on his former investigations relative to the digestion and assimilation of amyloid substances, draws the following inferences:—1. The alkalies normally contained in the blood and in the animal fluids are the principal agents of the digestion and the assimilation of saccharine and amyloid substances. 2. Starchy aliments are in all animals transformed into glucose by the agency of the animal diastose, whereby they become absorbable; this glucose, in order to become assimilable, is then transformed by the alkalies of the blood into new products, as kali-saccharic acid, formic acid, ulmin, &c., which bodies are all endowed with a very energetic disoxygenizing power, and probably destined to act as a counterpoise to the respiratory oxygenation. In a healthy subject, the usual alkalinity of the blood is amply sufficient for the transformation of the saccharine matter, but if this alkalinity be deficient, the transformation cannot take place; the sugar, being then neither decomposed nor assimilated, spreads itself over the economy, becomes a foreign body, and is, as such, cast off, not only by the kidneys, but by all secreting surfaces, and then we have diabetes. The cause of this affection may therefore be traced to a defective assimilation of the sugar, through a want of alkalinity in the animal economy. Human blood is naturally alkaline: we constantly introduce into our system acid elements, which would eventually predominate if they were not counterbalanced by especial secretions—viz., the perspiration and urine. So that a healthy man has one kind of secretion always yielding an acid reaction—viz., the perspiration and urine, and another kind, with alkaline properties—viz., the saliva, tears, and fæces. So long as these secretions retain their normal chemical nature, the due balance of acid and alkaline principles in the economy is kept up; if they all become acid, we, of course, conclude on a want of alkalinity, and *vice versa.* The former state may be brought on—1st, by the ingestion of acids themselves; 2d, by exclusively azotized food. Meats, owing to the albuminoid substances to be found in them, contain much sulphur and phosphorus, and those bodies generate sulphuric and phosphoric acids by their combustion within the economy. These agents get diffused through the fluids, saturate at first the alkaline bases they meet with, and at last predominate. 3d. The want of the perspiring action of the skin, which is intended to throw off acids from the economy. Thus I shall be able to show, says M. Mialhe, that by using means opposed to these three causes mentioned above, we can bring back the economy to its normal state, and excite in it a series of new phenomena. So that it appears possible—1st, to modify, as we please, the fluids effecting nutrition either in animals or vegetables, and obtain a proof of the reality of this modification by the examinatiou of the secretions; 2d, to invert, by means of the food or medicines, the natural order of the assimilating functions, and thus give rise to new phenomena, which change the normal products of the organism; 3d, to control, on the other hand, the accidental disturbance of the organism, re-establish the integrity of its functions, and thus re-constitute life and health. By applying these consequences to the diabetic affection, the author proposes to restore the vitiated humours to their normal standard, and re-establish the natural order of the assimilating functions, by introducing into the economy the alkali which is wanting, and expelling the acids which predominate by the use of alkalies and sudorifics. When diabetes is produced by a prolonged ingestion of acid substances, unaccompanied by suppression of the perspiration or deep alterations of the organism, the cure by alkalies may, in some degree, be instantaneous. He cites the case of a gentleman who exhibited all the symptoms of diabetes, seemingly brought on by an excess of acidulated drinks during the hot summer of 1847. Five drachms of bicarbonate of soda, with seventy-five grains of calcined magnesia, and two and a half bottles of Vichy water per diem, sufficed to remove all the symptoms in eight or ten days.

Now we are ready to do justice to M. Mialhe's indefatigable zeal in organo-chemical investigations; but it must be confessed, that a single case is far from having sufficient weight for establishing a new theory. The author has the foible of most labourers in the field of physiological chemistry. He will needs consider the organism as a regular laboratory; for him the stomach is a still, the liver a filter, the lungs a furnace, and the skin an evaporating apparatus, &c. MM. Martin, Solon, Bussy, and Rayer, will report upon this paper, of which the above is but an abstract; they will inform us whether the proofs alleged are satisfactory, for hitherto we see but an ingenious grouping of assertions and deductions. We shall therefore recur to the subject, but cannot leave it without remarking with what facility and readiness M. Mialhe harmonizes chemical effects. For instance: when speaking of the new products resulting from the action of alkalies upon the sugar, he mentions that these products are highly disoxygenizing, and immediately adds, that they, according to all probability, serve as a counterpoise to the respiratory oxidation. This appears rather a hasty conclusion. Another passage, which is strongly indicative of the exclusiveness of the author's chemical views, is that wherein he mentions the development of sulphuric and phosphoric acids, attributed to the ingestion of phosphorus and sulphur in meat. These acids, according to him, gradually saturate the alkaline bases in the fluids, and finally predominate. Here, again, we have a chemical reaction, which is simple and natural enough out of the body, assumed as taking place, just in the same way, in the mysterious recesses of our capillaries. Sir James Murray regards the human frame as a Leyden jar; Dr. Parkin, as a gasometer; M. Mialhe, as a laboratory! The humorists, solidists, mechanists, and chemists of old were hardly more wedded to peculiar views.—*London Lancet.*

